# A family based study on T-C transition polymorphism in Cyp17a1 gene in Indian children

**DOI:** 10.1186/1755-8166-7-S1-P63

**Published:** 2014-01-21

**Authors:** Sukanya Gayan, Abid Ali, Rajeev Kumar Pandey, Minu Bajpai

**Affiliations:** 1 Department of Pediatric Surgery, All India Institute of Medical Sciences, New Delhi, India

**Keywords:** Congenital adrenal hyperplasia (CAH), restriction fragment length polymorphism (RFLP), Cytochrome P450 17A1 (CYP17A1)

## Background

Congenital adrenal hyperplasia (CAH) or 46, XX DSD is a result of a defect in the P450 adrenal enzymes responsible for the conversion of progesterones to glucocorticoids and mineralocorticoids. This syndrome affects both males and females but causes ambiguous genitalia only in females. Mutations in the CYP21 cause 90% of cases of CAH. The remainder of CAH cases is distributed among deficiencies of P450c11, CYP17 or steroid acute regulatory protein, depending on the ethnic origin of patients. Rarely, 46, XX, DSD can result from exposure to exogenous androgens such as those given in the past to prevent loss of pregnancy.

The present study was conducted to replicate a family based correlation of C to T transition polymorphism in Congenital adrenal hyperplasia (CAH).

## Materials and methods

A total of 60 samples (20 families) within a period of one year (October 2012 – 2013) associated with CAH, were collected from patient visiting the Out Patient Door (OPD) facility of the department of Paediatric Surgery, All India Institute of Medical Sciences (AIIMS). Genomic DNA was isolated from peripheral blood leukocytes of twenty patients and their family members using the phenol - chloroform method. Polymerase chain reaction–restriction fragment length polymorphism (PCR-RFLP) was used to detect the polymorphism in CYP17A1 using restriction enzyme, MSPA1.

## Results

This study revealed no association between CAH risk and CYP17A1 gene in Indian children.

## Conclusion

Our results do not suggest a role of CYP17A1 as a high susceptibility gene for Congenital adrenal hyperplasia in Indian family.

